# POLICY ATTENTION NEEDED TO IMPROVE DIRECT CARE WORKER WAGES AND OTHER CHALLENGES

**DOI:** 10.1093/geroni/igac059.986

**Published:** 2022-12-20

**Authors:** Denise Tyler, Robyn Stone

## Abstract

Direct care workers (DCWs), including nursing assistants, home health aides, and personal care assistants, play an essential role in the health and well-being of over 20 million Americans who receive long-term services and supports (LTSS) at home, in nursing facilities, and other settings. In 2020, nearly 4 million DCWs supported older adults and people with disabilities in completing self-care and other daily tasks. Their efforts require considerable technical and interpersonal skills, but these essential workers receive low pay and rarely receive benefits. The COVID-19 pandemic has increased the attention paid to DCWs in the media and among policymakers. This symposium presents the results of three studies conducted by RTI International and the Office of the Assistant Secretary for Planning and Evaluation (ASPE) focused on DCWs. First, we present the results of a study examining how the wages of DCWs compare to the wages of other entry-level workers across all 50 states and report the effect of state policies aimed at improving DCW wages. Second, we report findings from a study exploring the experiences of home care aides during the pandemic as well as the federal and state policies implemented to assist these DCWs during the pandemic. Finally, we present the results of a study that assessed the effect of the COVID-19 pandemic on DCW staffing in nursing facilities. Together these studies suggest that more policy attention is needed to improve direct care work and attract the millions of additional DCWs that are expected to be needed in coming years.

